# Rheological and Technological Aspects in Designing the Properties of Shear Thickening Fluids

**DOI:** 10.3390/ma14216585

**Published:** 2021-11-02

**Authors:** Radosław Żurowski, Paweł Falkowski, Justyna Zygmuntowicz, Mikołaj Szafran

**Affiliations:** 1 Faculty of Chemistry, Warsaw University of Technology, 3 Noakowskiego Str., 00-664 Warsaw, Poland; pawel.falkowski@pw.edu.pl (P.F.); szafran@ch.pw.edu.pl (M.S.); 2 Faculty of Materials Science and Engineering, Warsaw University of Technology, 141 Wołoska Str., 02-507 Warsaw, Poland; justyna.zygmuntowicz@pw.edu.pl

**Keywords:** ceramic–polymer composites, intelligent materials, protective properties, rheological properties, shear thickening fluids

## Abstract

This work focuses on shear thickening fluids (STFs) as ceramic–polymer composites with outstanding protective properties. The investigation aims to determine the influence of raw material parameters on the functional properties of STFs. The following analyses were used to characterize both the raw materials and the STFs: scanning electron microscopy, dynamic light scattering, matrix-assisted laser desorption/ionization time-of-flight, chemical sorption analysis, rheological analysis, and kinetic energy dissipation tests. It was confirmed that the morphology of the solid particles plays a key role in designing the rheological and protective properties of STFs. In the case of irregular silica, shear thickening properties can be obtained from a solid content of 12.5 vol.%. For spherical silica, the limit for achieving shear thickening behavior is 40 vol.%. The viscosity curve analysis allowed for the introduction of a new parameter defining the functional properties of STFs: the technological critical shear rate. The ability of STFs to dissipate kinetic energy was determined using a unique device that allows pure fluids to be tested without prior encapsulation. Because of this, it was possible to observe even slight differences in the protective properties between different STFs, which has not been possible so far. During tests with an energy of 50 J, the dissipation factor was over 96%.

## 1. Introduction

The high expectations of society in almost every industry branch have become a reason for the substantial progress of science [1]. The interdisciplinary approach of researchers worldwide, and thus the synergy of many knowledge disciplines, has contributed to the establishment of a new scientific field: composite materials. Materials with unusual features are the result of combining the properties of various components that create them. In the 21st century, composite materials are present in almost every aspect of our lives, especially in application areas where their innovative and “intelligent” character dominates. Intelligent materials, also known as smart materials, can stand alone or constitute a larger functional structure or a more significant structural element. They can be defined as materials capable of responding to external stimuli by significantly changing their properties for the desired and effective response to these stimuli.

An excellent example of intelligent ceramic–polymer composite materials are shear thickening fluids (STFs). STFs are classified as non-Newtonian fluids, which means that their viscosity strongly depends on the applied shear forces; after exceeding the critical shear rate, their viscosity rises rapidly [2,3]. Initially, this feature was treated mainly as a disadvantage, especially in technological processes [4]. The resistance of this type of fluid can cause difficulties in mixing or flow. Consequently, this has often led to a lack of patency in industrial installations.

However, many years of research on the shear thickening phenomenon have shown that these materials have great potential. The application of sudden force to an STF increases the internal friction and blockage of material flow. For these reasons, the application of external forces causes a change in shear thickening fluids’ internal structure, which temporarily exhibits the properties of a solid. This internal remodeling of the material is responsible for the fact that STFs have an increased ability to dampen vibrations or shocks and dissipate the impact of energy [3]. Importantly, this process is reversible—when applied forces stop working, the fluid returns to its original state [5]. 

Due to their unique properties, shear thickening fluids can be used in many applications, as demonstrated by numerous scientific reports. The main application of STF composites is in the production of protective materials, both those intended for athletes and for uniformed services or special forces [6,7]. Work in this area focuses mainly on the reinforcement of high-strength p-aramid fabrics [8,9,10] or ultra-high molecular weight polyethylene (UHMWPE) fibers [11,12] to produce stab-proof and bulletproof vests. The obtained samples in this study were subjected to resistance tests in quasi-static, low, and high-velocity conditions to confirm the positive effect of this STF application [13,14,15,16]. 

There are also numerous examples of shear thickening fluids applied in the design of protective materials for athletes [17], such as shin guards [18,19], hip pads [20], or bandages impregnated with STF, to protect against sprains and dislocations [21]. 

Another application of shear thickening fluids related to the protection of the human body is their use in surgical gowns, surgical gloves, and other clothing worn by medical personnel, especially those exposed to the risk of puncture with sharp instruments during medical procedures [22]. Another medical application of STFs is the Allen-constructed limb restraint. The main element of this invention is an STF-based mechanism that prevents sudden limb movements of people who are currently undergoing treatment for injuries [23]. 

Materials exhibiting a shear thickening effect are also useful in mechanical, civil, space, and energy engineering. STFs have been introduced in the process of creating seismic dampers [24], vehicle brakes [25], vehicle dampers [26], train carriage joints [27], elements to dampen high-frequency vibrations occurring in pipe connections or elements of airplanes [28], protective suits for astronauts [29], elements of the outer shells of spacecraft [6,30], or lithium-ion batteries [17,31].

Shear thickening fluids are obtained mainly by the mechanical homogenization of solid-phase particles in a liquid, usually an organic matrix. However, there are also some cases where STFs are obtained by sonochemical methods [32]. Silica is a primary choice as a solid phase for STF production [16,33,34], but other raw materials such as corn starch [35], calcium carbonate [36], titanium dioxide [37], polymethyl methacrylate [38], polystyrene [39], or ethyl polystyrene acrylate [40] are also used for this purpose. The continuous phase of an STF is usually ethylene glycol [41,42], poly(ethylene glycol) [32,43,44], propylene glycol [41], or poly(propylene glycol) [7,45,46,47]. There are also reports on the application of glycerin [48], ethanol [37], and even water [49]. The scientific literature also provides information on the many modifiers of the rheological properties of STFs and other fluids used in industry. These modifiers can influence the viscosity in a significant way, even when used in small amounts [50]. The additives used include halloysite [51], aluminum oxide [52], cellulose nanofibers [53], silicon carbide [52], boron carbide [52], carbon nanotubes [18], graphene and graphene oxide [54,55], expanded graphite [56,57], or poly(propylene glycol) diacetate [58]. Often, the material property modifier can also be silica itself, e.g., in multiphase fluids [59]. In addition to external parameters such as temperature [42,60] or humidity [5,61], the types of raw materials used and their volume fractions are the main factors determining the rheological properties of an STF. Table 1 summarizes the most important factors related to raw materials with a brief description of their impact on the shear thickening effect.

There are many proposals for shear thickening mechanisms in the scientific literature. The most widespread are the Reynolds mechanism, the Order–Disorder Transition (ODT) theory [70], the hydrocluster theory [71,72], the flocculation theory [73], and the collision theory [74]. An interesting look at the phenomenon of shear thickening can also be seen in a publication by Peters et al. [75]. Which model can be used to explain the phenomenon of shear thickening strongly depends on the specific fluid—the raw materials used and their proportions. All the mechanisms mentioned are described in the cited publications, therefore we will not discuss them in more detail. 

Scientific papers dealing with shear thickening fluids can be roughly divided into two groups. In the first group, we can find numerous reports describing the physicochemical processes taking place inside the STF structure, theories explaining the shear thickening mechanism, or the general rheological characteristics of these materials depending on many factors that were mentioned above. However, there are no reports describing STFs in a more holistic way, i.e., trying to explain not only the influence of certain factors on the rheological properties of shear thickening fluids but also how these properties affect their ability to dissipate kinetic energy. To put it briefly, it is relatively easy to find, in scientific articles, a relationship between the composition and the rheological properties of STFs, but difficult to find a relationship between the rheological and protective properties of pure STFs. The reason for this is probably the lack of equipment designed to measure the energy dissipation capacity of liquid materials. 

The second group of publications concerns attempts to implement shear-thickening fluids to produce more complex structures or prototypes which dissipate kinetic energy. STFs are usually utilized by filling porous, three-dimensional structures (e.g., various types of foam or warp-knitted spacer fabrics) or silicone forms, or play a role as an impregnating agent for woven, knitted, and other flat composite mats made of high-strength materials, such as p-aramid or UHMWPE. The main methods for STF deposition are impregnation, dipping extrusion, spray gun, brush, and filling [76]. This group of publications includes reports on the positive impact of an STF’s presence in the structure of more complex composite materials, including prototypes for knife- and bulletproof vests as well as protective gear for sports. A complementary component of these publications is considerations about how these fluids work in the system. In this group of publications, we can also find studies in which shear thickening fluids of different compositions and thus different rheological properties were used to produce samples for drop or ballistic tests. However, in these cases, the response of the pure fluids to the given stimulus was not measured. The recorded responses were not from the STF but from the larger structure containing this STF. Thus, it is not possible to directly relate the rheological properties of shear thickening fluids to their actual energy dissipation capacity. According to the authors, the relationship between these two parameters is one of the key issues on the way to a fuller understanding of the subject of shear thickening fluids. The ability to combine the rheological and protective properties of STFs will also be useful in designing their compositions. Because of this knowledge, the performance properties of the produced fluids could be tailored to specific requirements as much as possible.

The development of STFs and the exploration of knowledge in this area are still very important and necessary. Interest in these materials is growing every year. This is evidenced by the constantly growing number of scientific papers dealing with this issue. In addition, there are established companies in the world that are strictly focused on the production and implementation of shear thickening fluids in industry. An example is the American company STF Technologies LLC [77]. It is worth noting that this is despite the fact that obtaining and processing shear thickening fluids on a larger scale is extremely difficult, due to their non-Newtonian flow nature.

The aim of this paper was to correlate the effect of particle shape on shear thickening properties with the direct response of the designed fluids to kinetic impact. This was made possible thanks to extensive rheological measurements as well as impact energy dissipation tests, with the use of a specially designed and constructed piece of equipment called a drop test machine. It must be underlined that for these tests, pure STF was applied without any special packaging, injection, or impregnation into other structures. Due to this, a broader view of the shear thickening effect and its practical aspects was made possible.

Due to such great interest in the subject of STFs and serious attempts to implement these materials in the industry, it was decided that this paper would focus on shear thickening fluids of the simplest possible composition. Two-component systems based only on silica and poly(propylene glycol) were selected. From a technological point of view, materials that are simple, but at the same time exhibit unique properties, have the greatest implementation potential. Two types of the most common silica powders (fumed and sol-gel silica) were used to design and obtain STFs. It is worth underlining that the method of producing these raw materials significantly determines their morphology. This has a colossal impact on the possible composition of shear thickening fluids, as well as on their functional properties.

## 2. Materials and Methods

### 2.1. Materials

Two types of silica powder, differing in production method and particle morphology, were used as the solid phase of the shear thickening fluids. KE-P10 is an amorphous silica made by the sol-gel Stöber process [78]. According to the manufacturer’s specification (Nippon Shokubai, Tokyo, Japan), the powder particles have a spherical shape, and their size is in the range of 100–200 nm. The purity of silica is ≈90%, and the main impurities are the methyl and butyl alcohols remaining after the production process. 

The second ceramic powder was fumed silica (SF), supplied by Sigma-Aldrich, St. Louis, MO, USA. According to the information provided by the manufacturer, the nanometric (7–14 nm) powder particles have an irregular shape and form agglomerates with a size of 200 to 300 nm. 

Poly(propylene glycol) with an average molar mass of 425 g/mol, provided by Sigma-Aldrich, St. Louis, MO, USA, was utilized as a carrier fluid in all STFs obtained. 

MM 922 molding silicone (ACC Silicones, Bridgwater, Somerset, UK) was used to produce composite samples of STF–silicone for the first kinetic energy dissipation tests. MM922 silicone has a density of 1.26 g/cm^3^, a hardness of 22 °ShA, a tensile strength of 3.64 MPa, and an elongation at a break of 497%. The cross-linking time initiated with the MM CAT B5 catalyst (ACC Silicones, Italy) was 8–12 h.

### 2.2. Characterization of Raw Materials

#### 2.2.1. Characterization of the Solid Phase

The specific density of the silica powders was determined using the AccuPyc 1340 helium pycnometer (Micromeriticis, Norcross, GA, USA). The bulk density was evaluated by loosely filling a vessel with a known volume and determining the mass of silica.

The investigation of the morphology of the silica particles was possible thanks to observations made using the scanning electron microscope (Carl Zeiss, Oberkochen, Germany). Additionally, the particle size and particle size distribution in the water suspension were determined by the dynamic light scattering method, using the Zetasizer Nano ZS particle size analyzer (Malvern Panalytical, Malvern, Worcestershire, UK).

The specific surface area of the powders was defined by the nitrogen adsorption and desorption method, using the Brunauer-Emmett-Teller (BET) isotherm on the ASAP 2020 chemical sorption analyzer (Micromeriticis, Norcross, GA, USA).

#### 2.2.2. Characterization of the Continuous Phase

The viscosity of the carrier fluid was determined using a KinexusPro rotational rheometer (Malvern Panalytical, Malvern, Worcestershire, UK) equipped with plate–plate geometry. Measurements were carried out at a temperature of 20 °C. The measurement gap was 0.3 mm.

The molecular weight distribution and dispersity of the PPG were evaluated by the MALDI ToF (matrix-assisted laser desorption/ionization time-of-flight) sample ionization technique using an ultrafleXtreme mass spectrometer (Bruker, Billerica, MA, USA); 2,5-dihydroxybenzoic acid was used as the matrix.

### 2.3. Preparation of Shear Thickening Fluids

Two series of non-Newtonian fluids with two components (KE-P10 or SF silica and PPG 425) were prepared. All suspensions were produced by mixing dry silica powder with PPG 425 using the R50D mechanical stirrer (Ingenieurbüro CAT, Ballrechten-Dottingen, Germany). The preparation process was carried out until the samples were completely homogeneous. Depending on the fluid composition, the mixing time ranged from 45 min to 4 h.

### 2.4. Characterization of the Rheological Properties of STFs

The rheological properties of all fluids obtained were determined using a KinexusPro rotational rheometer (Malvern Panalytical, Malvern, Worcestershire, UK) working in plate–plate geometry. The measuring gap was 0.7 mm. All tests were carried out under isothermal conditions. The temperature of 20 °C was stabilized with a Peltier module.

The rheological characteristics of shear thickening fluids include both dynamic viscosity tests as a function of the shear rate and oscillatory and thixotropic measurements. Regardless of the type of measurement performed, each was preceded by a pre-shearing procedure. After loading the analyzed fluid, the temperature was first stabilized for 60 s. The sample was then sheared for 120 s at a shear rate of 0.1 s^−1^, followed by a 5 min relaxation period of the material, and then an actual test. 

In order to determine the viscosity curves of the obtained fluids, measurements were taken with a logarithmically increasing shear rate in a range from 10^−1^ to 10^3^ s^−1^.

The viscoelastic properties of the selected STF were evaluated by dynamic oscillatory measurements. In the first step, the linear viscoelastic region (LVER) was determined for the specific fluid. For this purpose, an amplitude scan with amplitude deformation in a range from 0.1 to 500% was performed at a constant frequency (1.6 Hz ≈ 10 rad/s). In the next step, the fluid was treated with a variable frequency of 10^2^ to 10^−1^ Hz with a constant value of strain belonging to the LVER. Finally, the dependences of the G′-storage (elastic) modulus and G″-loss (viscous) modulus as a function of frequency were plotted. 

The G′-storage modulus is responsible for the elastic part of the viscoelastic behavior of the material; in other words, it represents the stored deformation energy. Therefore, G′ quasi describes the solid-state behavior of the material. 

The G″-loss modulus is responsible for the viscous part of the viscoelastic behavior of the material; in other words, it represents the deformation energy dissipated via the internal friction during flow. Therefore G″ quasi describes the liquid-state behavior of the material.

To determine the thixotropic properties of the selected STFs, interval tests were performed according to the shear rate jump method. Each test consisted of 3 stages:

- A simple shear of the sample with a shear rate of 10^−1^ s^−1^ for 100 s;
- A rapid increase in the shear rate to 100 s^−1^ for 3 s;
- A simple shear of the sample with a shear rate of 10^−1^ s^−1^ for 10 min.

During the second stage, the authors wanted to imitate the sudden application of large force to the system, imitating, for example, an impact. In such a case, the time could be even shorter, but taking into account the measuring inertia of the rheometer, it was extended to 3 s.

**Figure 1:**
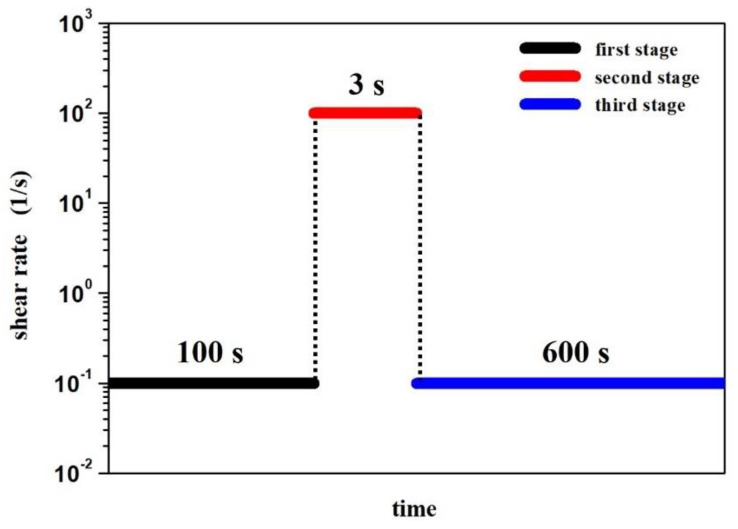
The scheme of the 3-step interval test.

The measurement procedure is presented schematically in Figure 1. On the basis of the recorded data, the dependence of the dynamic viscosity as a function of time was plotted. These measurements allowed us to observe the rheological response of the material to the rapidly applied high shear forces, and then to rebuild the fluid’s structure.

### 2.5. Kinetic Energy Dissipation Tests

#### 2.5.1. Kinetic Energy Dissipation Tests with Silicone–STF Samples

**Figure 2:**
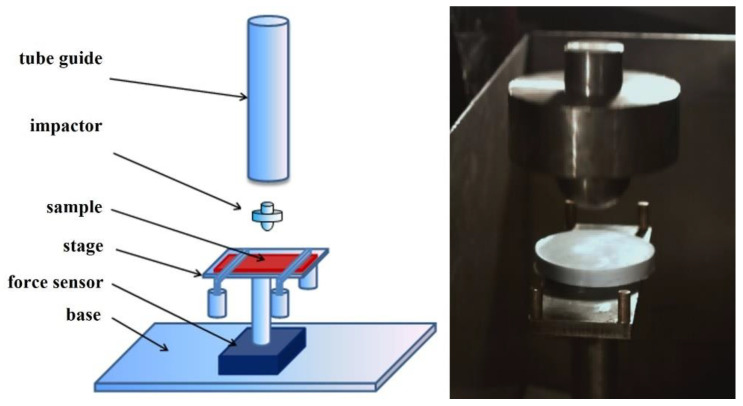
The scheme of the drop test machine and a frame from a high-speed camera, showing an element hitting the sample.

The first measurements of kinetic energy dissipation were performed with a typical drop test machine, which was designed and constructed on the basis of the British standard BS 7971-4: 2002. The device scheme is presented in Figure 2.

**Figure 3:**
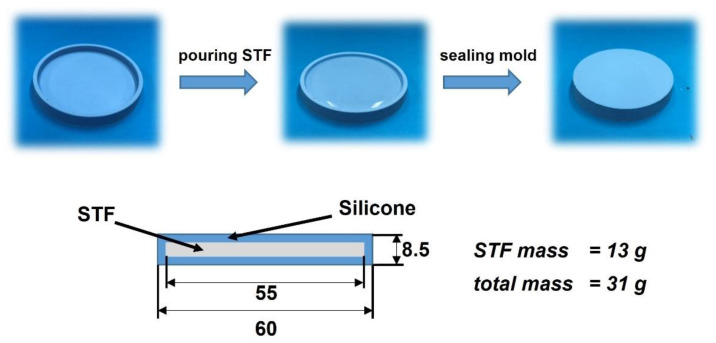
The scheme of obtaining a silicone–STF composite material and the dimensions of a single sample designed for drop testing.

The silicone–STF composite samples for the drop tests consisted of 13 g of the selected shear thickening fluid enclosed in an 18 g silicone mold. A simple scheme for obtaining a silicone–STF composite material, as well as the dimensions of a single sample, is shown in Figure 3.

During the tests, the silicone–STF samples were placed on the stage, under which the force sensor was located. Then, a 2 kg impactor was dropped onto the sample (Figure 2). The impact energy of 5 J was controlled by adjusting the height of the striker. The difference in the recorded force value for the reference measurement (without the sample) and the actual measurement (with the sample) allowed the percentage determination of the degree of dispersion of the impact force. The experiments were additionally recorded with a HiSpec high-speed camera with a speed of 1000 frames per second. It should be underlined that measurements conducted in this way allow one to obtain a response not from a pure STF, but from a composite containing a specific fluid. Despite the fact that the mass of the silicone mold in each sample was the same, its presence significantly influences the value of the force passing through the tester, as recorded by the sensor. This fact should be taken into account when analyzing the results.

#### 2.5.2. Kinetic Energy Dissipation Tests in the Rod–Cup Measuring System

**Figure 4:**
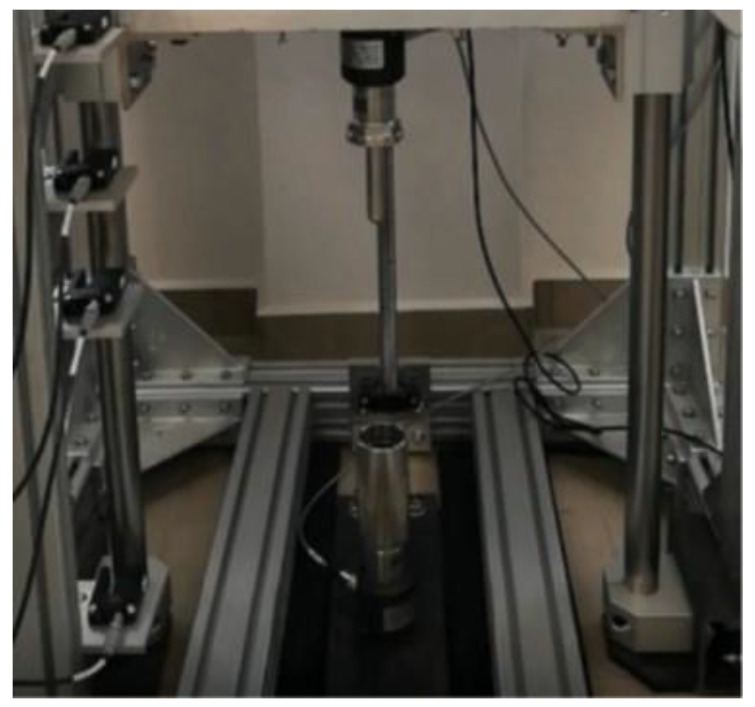
The photography of the drop test machine with a rod-cup measuring system.

The limitations in conducting kinetic energy dissipation tests using only fluid materials were overcome by using a drop test machine with a rod–cup measuring system (Figure 4). The unique design of the device allows for the measurement of shear thickening fluids without the need for prior encapsulation or packaging.

In order to conduct a single drop test, the following activities were performed:

1. A bottom-threaded steel cup was filled with a specific shear thickening fluid. The fluid column height equaled 80 mm and its mass was approximately 145 g;
2. The cup filled with STF was screwed into the force sensor attached to the bottom plate;
3. The appropriate height of the traverse on the guideway was set;
4. The traverse, with the impactor, was dropped onto the STF;

The internal dimensions of the steel cup were:

- Sample (fluid) space: *h* = 80 mm and Ø = 40 mm;
- Space above the sample (fluid): *h* = 20 mm and Ø = 50 mm.

**Figure 5:**
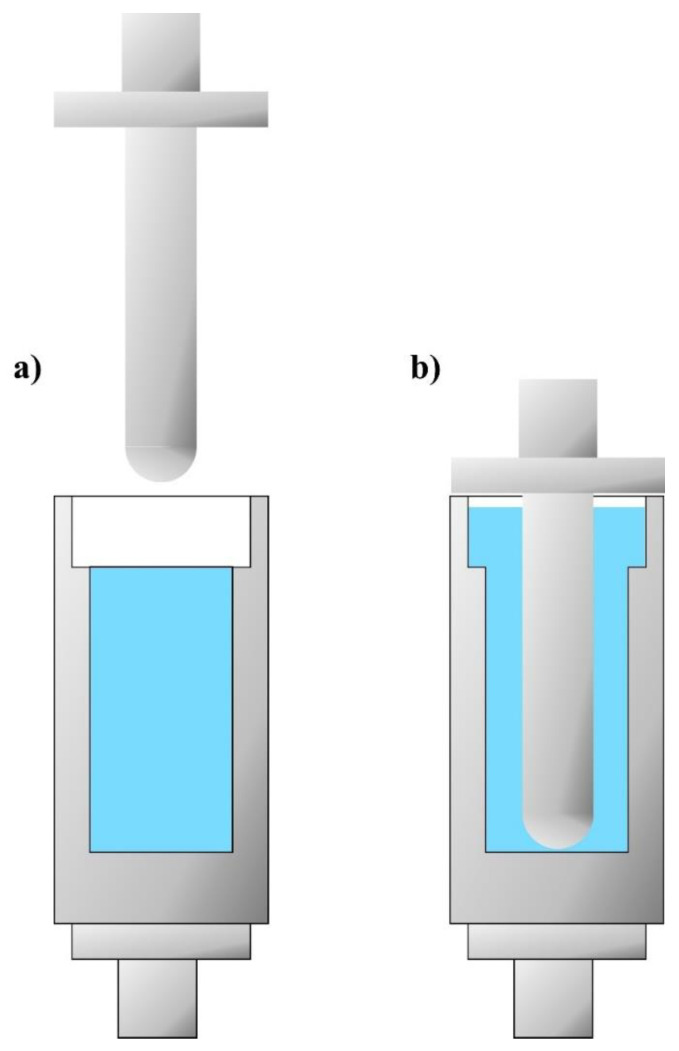
Scheme of fluid behavior in the cup: (**a**) before and (**b**) after the rod impact during the drop tests.

This design of the cup provided a sufficient amount of space for the fluid displaced by the rod during impact and prevented it from escaping from the cup (Figure 5).

The impactor was shaped like a spherically ended rod, 100 mm in length and 20 mm in diameter. The impact energy was equal to 50 J. It was controlled by adjusting the height of the falling traverse, the mass of which was 17 kg. The force sensor data collection frequency during the tests was 10 kHz.

The result of the measurements was the value of the force recorded by the sensor located under the sample. The lower the value of the recorded force, the more energy was dissipated by the specific STF.

## 3. Results and Discussion

### 3.1. Characteristics of the Materials Used

**Figure 6:**
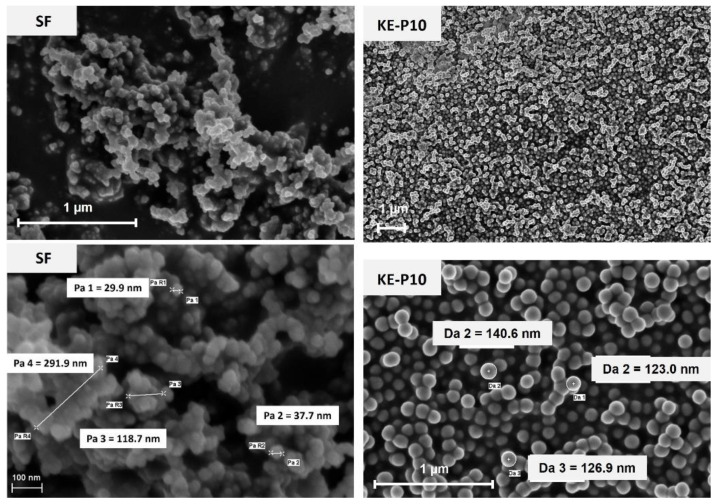
SEM images of SF and KE-P10 silica powders. Pa and Da marks show examples of single silica particle diameters and agglomerate size measurements for SF and KE-P10, respectively.

The physicochemical properties of the materials used are summarized in Table 2 and Table 3. SEM images of the KE-P10 and SF silica powders are shown in Figure 6.

The SEM images of the KE-P10 particles show that the morphology of these particles is spherical, with an average particle size of around 126 nm. This powder also visibly shows a slight tendency for agglomeration; however, inside the agglomerates you can easily distinguish individual particles (clear borders between particles), suggesting that the bonding forces within the agglomerates are quite weak. These observations are supported by DLS measurements, in which the measured average particle size was 127 nm with a fairly narrow monomodal distribution. This means that the agglomerates are broken up with ease during the preparation (sonication) of the silica suspension before DLS measurement.

The shape of the other silica used (SF) is rounded, but the surface is quite irregular, with an average particle size claimed by the manufacturer to be in the range of 7 to 14 nm. However, the SEM images (Figure 6) show that the average particle size is rather in the range of 30 to 40 nm and the powder shows a strong tendency for agglomeration. Due to this agglomeration, the measured particle size by the DLS method is ca. 218 nm, roughly corresponding to agglomerates composed of 160–380 particles.

Despite small differences, the physicochemical parameters of the powders used are consistent with the characteristics given by the manufacturers. Only in the case of KE-P10’s bulk density was a significant difference found (Table 2); however, this might be caused by mechanical pressing during transportation and long storage in soft plastic bags.

The PPG 425 used as the dispersing fluid shows a Newtonian character in the tested shear rate range (up to 10^3^ s^−1^), and its viscosity measured at T = 20 °C was 0.08 Pa∙s. The number average molecular weight, measured by MALDI ToF, was Mn = 498. This analysis also confirmed the unimodal molecular weight distribution of the oligomer. These data are broadly in line with the specifications provided by the manufacturer.

### 3.2. Effect of Silica Morphology on the Rheological Properties of STFs

**Figure 7:**
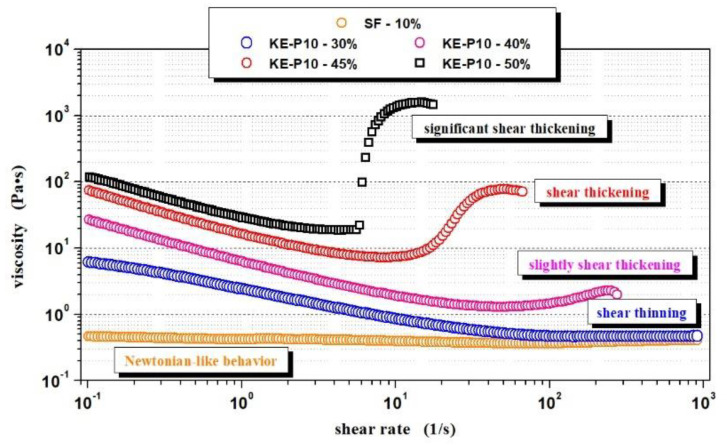
Exemplary fluid viscosity curves showing different rheological behaviors depending on composition. The fluids were made from PPG, with an average molecular weight of 425, and KE-P10 or SF silica with various concentrations of the solid phase in the system. Measurements were carried out at 20 °C.

To determine the effect of silica morphology on the rheological properties of non-Newtonian fluids, two series of samples with SF or KE-P10 silica were prepared. In both series, PPG 425 was used as dispersing medium. The samples in a given series differed in the concentration of the solid phase. Table 4 summarizes the rheological behavior of the prepared samples, while Figure 7 shows examples of all types of rheological behavior observed during the measurements.

where:

- Newtonian-like behavior—the fluid displayed a relatively constant viscosity at a shear rate range of 10^−1^ to 10^3^ s^−1^;
- Shear thinning—between a shear rate of 10^−1^ and 10^3^ s^−1^, the viscosity of the fluid decreased with increasing shear rate;
- Slight shear thickening—between a shear rate of 10^−1^ and 10^3^ s^−1^, a small increase in viscosity was observed; however, the increase in viscosity was no greater than 50 Pa∙s;
- Shear thickening—between a shear rate of 10^−1^ and 10^3^ s^−1^, an increase in viscosity was observed and was greater than 500 Pa∙s;
- Significant shear thickening—between a shear rate of 10^−1^ and 10^3^ s^−1^, a sudden and sharp increase in fluid viscosity was observed and was greater than 500 Pa∙s;
- Not possible to obtain—the solid phase concentration was too high and it was not possible to prepare such a sample;
- Homogenous fluid—it was possible to obtain the homogenous dispersion of silica in PPG; however, the viscosity was too high for reliable measurements of such fluid with a rotational rheometer;
- X—the fluid with this composition was not prepared.

The results in Table 4 show that the minimal concentration of irregular SF silica required to obtain a non-Newtonian STF should exceed 12.5 vol.%. At 20 °C, fluids based on PPG 425 with a lower content of SF show shear-thinning behavior which is very close to Newtonian behavior—the viscosity is almost independent of the shear rate. It is also worth noting that the maximum content of the solid phase that could be added to the SF–PPG 425 system was only 22.0 vol.%. Despite such a relatively low amount of silica, the shear thickening effect of the material was more than 8000 Pa∙s.

On the other hand, the same volume fraction of KE-P10 silica dispersed in PPG resulted in a typical shear-thinned suspension, the viscosity of which decreased in the tested range of the shear rate, from 1.7 Pa∙s up to 0.3 Pa∙s. Furthermore, the minimal concentration of KE-P10 required for the fluid to begin to exhibit shear thickening properties is 40 vol.%—more than three times higher than in the case of irregular SF silica. The maximum possible volume fraction of KE-P10 silica able to be introduced into glycol PPG 425 was 55 vol.%. The fluid obtained was visually homogeneous; however, its viscosity was too high to be correctly measured by a rotational rheometer. These results correspond quite well to the data presented by Peters et al. [75] based on the example of corn starch dispersed in an aqueous solution of glycerol and CsCl. Despite the differences in the continuous phase, the critical volume fractions of the solid phase, causing the dilatation effect, as well as the maximum content of particles in the prepared fluids, are at a comparable level.

**Figure 8:**
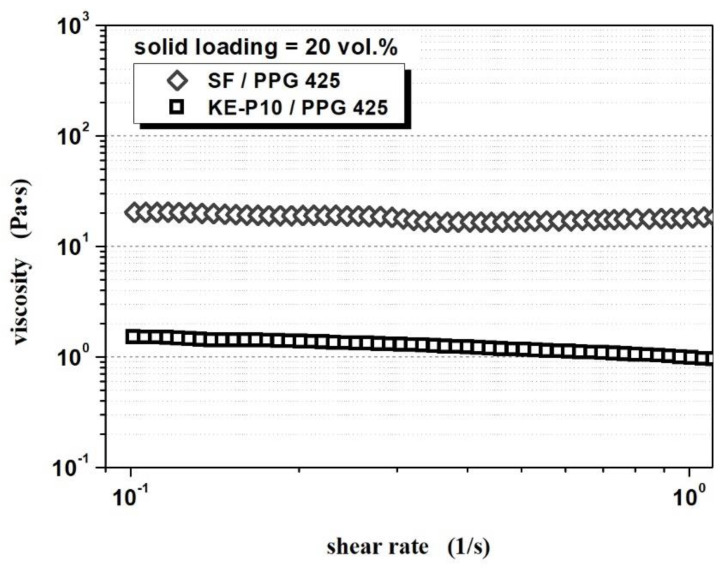
The viscosity of shear thickening fluids, based on spherical KE-P10 or irregular SF and poly(propylene glycol) with an average molecular weight of 425 in a shear rate range from 0.1 to 1.0 s^−1^. The solids contents in both cases were 20 vol.%.

A comparison of the rheological properties of fluids based on SF and KE-P10 silica, as well as a comparison of silica particle shapes (Table 4 and Figure 6), shows a significant influence of particle morphology on the rheological properties of an STF. The irregular shape of the particles and a strong tendency for agglomeration induce a thickening effect at much lower values of the solid phase. Furthermore, for the same volume fraction of the solid phase, fluids with SF silica show significantly higher initial viscosity (at low shear rates) than fluids with KE-P10 (Figure 8). These effects can be related to an increase in flow disturbance as the shape factor of the silica particles increases.

According to Barnes [4], Srivastava et al. [79], and Genovese [80], to obtain a shear thickening fluid with a relatively low solid content, it is more preferable to use a fibrous, needle-like, or irregularly shaped raw material than a spherical material, due to the much greater number of potential collisions of particles during shear, which will consequently block their flow at lower shear rates. The rotation of irregular particles during flow causes their blockage and clogging in disorganized clusters. This process is easy to observe and can be seen at a lower solid volume when the raw material has a larger shape factor. It should also be remembered that the higher surface area of the powder, in the case of irregularly shaped silica particles (SBET SF = 179 m^2^/g, SBET KE-P10 = 114 m^2^/g), also results in an increase in particle–particle and particle–glycol molecule interactions. Of course, the rheological behavior, apart from the particle shape and the specific surface area, is also significantly influenced by the size of the silica. SF powder, with a nominal particle size of 30–40 nm, is highly agglomerated in the system, and the size of the agglomerates is larger than the particle size of KE-P10. When considering the shear thickening phenomenon, irregular agglomerates block the possibility of flow more readily than spherical particles. Therefore, the shape factor was considered dominant and the main focus was placed on it.

Among the samples prepared for further analysis, two fluids were selected: KE-P10/PPG 425 with 50 vol.% of silica and SF/PPG 425 with 20 vol.% of silica. This choice was dictated by the fact that these fluids are characterized by a significant shear thickening effect at a similar level.

**Figure 9:**
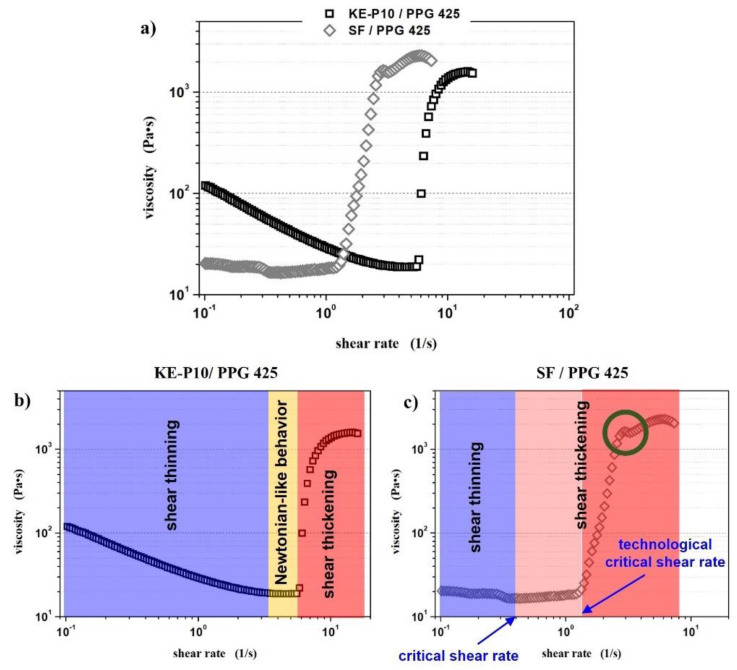
(**a**) Viscosity curves of shear thickening fluids based on spherical KE-P10 or irregular SF and poly(propylene glycol) with an average molecular weight of 425. The solids contents in the KE-P10 /PPG and SF/PPG fluids were 50 and 20 vol.%, respectively; (**b**,**c**) show the viscosity curves of both fluids, respectively, with an indication of areas with different rheological responses to the given rate of shear. The measurements were carried out at a temperature of 20 °C.

Figure 9 shows the viscosity curves of both selected fluids. Table 5 presents the values of the characteristic rheological values determined on the basis of the rheological measurements.

The profile of the viscosity curve of the fluid containing KE-P10 silica is three-stage, and the behavior of the sample at each stage is characteristic of a typical material thickened by shear [57,79]. In fluid based on silica and glycol, both at rest and during shear, poly(propylene glycol) molecules are adsorbed on the SiO_2_ surface. The main reason for this is the formation of numerous and relatively strong hydrogen bonds between the Si-OH silanol groups on the surface of the powder particles and the OH hydroxyl groups, which end the chain of PPG molecules (PPG generates strong hydrogen bonds with the silica surface [81]). In this way, the oligomer molecules surround the solid-phase particles, forming the so-called protective steric layer. The resulting repulsive forces, short and long-range, make it difficult for silica particles to aggregate into larger clusters in the STF, thus limiting flocculation and coagulation processes and contributing to the stabilization of the suspension [46,81]. To estimate the thickness of the PPG on the surface of the KE-P10 silica, additional measurements were performed using the dynamic light scattering method (DLS). The first measurements show that the average particle size of KE-P10 determined by this method was 127 nm. As was mentioned before, this result is almost identical to the particle size calculated on the basis of the SEM images. This might indicate that there was no agglomeration of KE-P10 particles in the suspension under the conditions of the measurements conducted. In the second measurement, the KE-P10 powder particles were dispersed in a deionized water–PPG 425 solution in a weight ratio of 4:1. As a result of the presence of PPG in the solution, the adsorption of the PPG molecules on the silica surface occurred, and a thin steric layer was formed. The measured average particle size of the silica molecules was 162 nm. Therefore, it can be concluded that the thickness of the steric layer formed by the PPG 425 oligomer particles around the KE-P10 silica particles was approximately 17 nm. Considering the relatively high volume of silica particles (50 vol.%) in the prepared fluid, there was probably an accumulation of interactions between SiO_2_ and SiO_2_, SiO_2_ and PPG, and PPG and PPG, such as Van der Waals interactions, dispersion interactions, or hydrogen bonding. It is also possible that, due to the Brownian motion in the dispersion, there could be collisions between the powder particles or that the movement could cause the distance between some particles to decrease, which would result in an increase in repulsive interactions. All of this contributes to the relatively high initial viscosity of the fluid (119.5 Pa∙s). The application of low shear force most likely breaks some of the hydrogen bonds, as well as the affecting the orientation of the silica particles and PPG chains (molecules) along the flow line, which reduces the viscosity of the fluid from an initial 120 Pa∙s to 19 Pa∙s at a shear rate of 3.1 s^−1^. This first phase is called the shear-thinning phase (Figure 9b, blue region). In the second stage (Figure 9b, yellow region), at a shear rate ranging from 3.1 to 5.8 s^−1^, the fluid shows typically Newtonian-like behavior, its components briefly interacting with each other in a state of thermodynamic equilibrium. A further increase in the shear rate causes the rapid multiplication of internal friction, observed macroscopically as a “jump” in viscosity (Figure 9b, red region), which is caused by the enhancement of hydrodynamic attractive forces, causing the formation of hydroclusters [72] and/or an inability to form an ordered state and a sharp increase in the number of collisions between silica particles [74].

However, it is unlikely that the KE-P10 silica particles would be unagglomerated in the analyzed fluid, mainly due to its large volumetric content, which is 50 vol.%. If the particles were totally separated and perfectly dispersed, the average surface-to-surface separation distance between particles (*SDP*) would be only ca. 2.9 nm, according to Equation (1) [82], and the effective volume fraction of the solid phase ($\varphi_{effect.}$), calculated from Equation (2) [83], would be greater than 1:

(1) $$SDP=d\left[{\left(\frac{1}{3\pi \varphi }+\frac{5}{6}\right)}^{\frac{1}{2}}-1\right]$$

where *d* is the particle diameter and φ is the volume fraction;

(2) $$\varphi_{effect.}=\varphi {\left(1+\frac{\delta }{r}\right)}^{3}$$

where φ is the volume fraction, *δ* is the thickness of the adsorbed layer, and *r* is the particle radius.

Therefore, in the analyzed shear thickening fluid, the average size of the silica “particles” is much larger due to their aggregation into dimers, trimers, tetramers, etc. As reported by Isobe et al. [82] the average particle size of alumina powder in an aqueous suspension strongly depends on its solids volume. They reported that the average size of TM-DAR Al_2_O_3_ particles (or, rather, agglomerates), measured by the electroacoustic method, does not change substantially up to 30 vol.% and is around 150 nm. As the solids content increases, a rapid increase in particle size is observed: for 40 vol.% it is over 200 nm and for 47.5 vol.% it is ca. 400 nm. It is obvious that the aqueous suspensions of alumina in the quoted publication differ significantly from non-aqueous STFs and the interactions between the components are probably of a slightly different nature. It must be also remembered that, to measure the mean particle size, diluted and usually sonicated aqueous suspensions are used. During STF preparation, strain and shear, induced by elements of the mixer, are too weak to break the bonds between particles and to homogeneously disperse them, especially at a high volume fraction of silica. However, simplifying the situation, and assuming a similar upward trend, the average particle size of the powder in the KE-P10/PPG 425 system with a silica volume content of 50% would be about 450 nm. In this case, the calculated *SDP* and the effective volume fraction would be 10.2 nm and 0.62, respectively, which are more probable results.

On the other hand, the SF/PPG 425 system has a solids content of 20 vol.%. According to Isobe et al., for such a low content of ceramic powder, no significant agglomeration should be observed, even if their particle size was 50 nm. Taking the average particle (agglomerate) size of SF silica as 218 nm (determined by the DLS method) and assuming that this size does not increase due to the low content of the solid phase, the *SDP* is over 36 nm, which is 3.5 times more than in the KE-P10/PPG 425 system. It can be stated that the bigger the distance between particles, the lower the rate of agglomerate–agglomerate interaction and friction in the fluid. That is why the initial viscosity of the fluid containing irregular silica is only 20.4 Pa∙s. The results of the rheological measurements for this STF show that the profile of the obtained viscosity curve (Figure 9c) is different than in the case of the fluid with KE-P10. It is not possible to distinguish three areas in which we observe, successively, shear thinning, Newtonian-like character, and shear thickening. At a shear rate range of 0.1 to 0.4 s^−1^, a slight reduction in viscosity takes place to the value of 16.5 Pa·s, after which point the fluid begins to thicken. In the beginning, the viscosity increases slowly as the shear rate ranges from 0.1 to 0.4 s^−1^, reaching 21.5 Pa∙s (Figure 9c, light red region). Above a shear rate of 0.4 s^−1^, a rapid increase in viscosity is observed (Figure 9c, red region). It must be noted that the “jump” in viscosity also takes place in two stages. After the first rapid increase in viscosity, up to 1640 Pa∙s, a slight decrease in viscosity was observed at a shear rate of 2.9–3.4 s^−1^ (Figure 9c, region marked by the green circle), followed by the next increase in viscosity; then, an increase in the sample’s internal friction was again observed. Such a two-stage curve course may be the result of a slight lack of homogeneity in the system, or, more likely, the result of the irregular shape of the silica particles, which may clog into larger clusters in a more unpredictable manner by interfering with each other. At some point, the shear force could cause the destruction and regrouping of the cluster-like structure, observed as a temporary decrease in viscosity. Finally, the fluid reaches a shear thickening effect of 2275 Pa∙s, which is 145% of the value measured for the STF containing 50 vol.% of KE-P10.

In the literature, the value of the shear rate beyond which the fluid viscosity begins to increase is called **critical shear rate** (${\dot{\gamma }}_{cr}$). According to this definition, in the case of the SF/PPG 425 system the critical shear rate is 0.4 s^−1^. However, from a technological point of view, a critical shear rate of 1.3 s^−1^ should be considered valid, because only after exceeding this shear rate does the viscosity of the system increase rapidly. Thinking about the practical use of STF materials, only a sudden and abrupt increase in viscosity ensures the proper response to the applied external stimulus. Therefore, it is postulated to distinguish the **technological critical shear rate** (${\dot{\gamma }}_{cr-T\;}$). The critical shear rate and the technological critical shear rate using the example of the SF/PPG 425 system are shown in Figure 9c.

**Figure 10:**
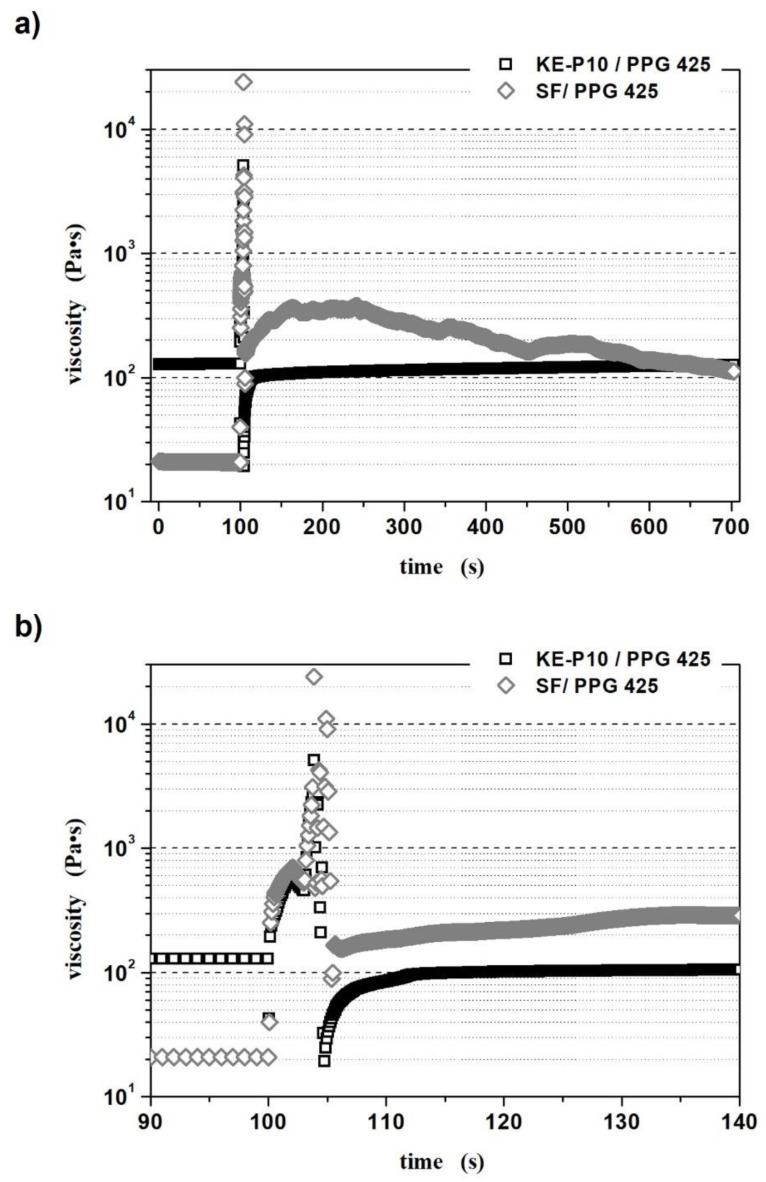
Dependence of dynamic viscosity as a function of time for the interval test with shear thickening fluids based on KE-P10 or SF silica and poly(propylene glycol) with an average molecular weight of 425: (**a**) in the range of 0–703 s and (**b**) in the narrower range of 90–140 s for a better analysis of the curve course. The solids contents of the fluids with KE-P10 and SF silica were 50 and 20 vol.%, respectively. The measurements were carried out at a temperature of 20 °C.

In order to perform a more precise analysis of the rheological properties of both fluids, the so-called interval test was conducted. The results of this test, i.e., the dependence of dynamic viscosity as a function of time, are presented in Figure 10.

In the first stage of measurement, at 0–100 s, the samples were subjected to a constant shear of 0.1 s^−1^. During this time, the viscosity of both fluids was relatively constant, although in the case of the sample containing KE-P10 silica, a slight increase in viscosity was noted—exactly 1.8 Pa∙s. A sudden increase in the applied shear force (a shear rate of 100 s^−1^ at 100–103 s) caused a rapid increase in total internal friction, which macroscopically manifested itself as a sharp “jump” in viscosity. Similar to the viscosity measurements, the rapid increase in viscosity is related to a structural reorganization and clustering process under shear, which increases the number of collisions between clusters/agglomerates/particles and leads to a significant increase in flow resistance. The maximum values of viscosity recorded at this stage of the measurement were 596 and 694 Pa∙s for the STFs based on KE-P10 and SF silica, respectively. In the third stage, a reduction in shear rate to the initial value of 0.1 s^−1^ caused a further, temporary increase in the viscosity of both materials, due to the inertia of the components and the progressive formation of even larger clusters of silica particles. For both fluids, with KE-P10 and SF silica, the highest viscosity values were observed at 103.9 s of the measurement and were over 5000 and 24,000 Pa∙s, respectively. After reaching these maximums, the samples began to return to their initial states and their viscosity began to drop rapidly. Interestingly, the reorganization of the structures of both these systems occurred in completely different ways.

The viscosity of the fluid with KE-P10 silica dropped to 19 Pa∙s at 104.8 s of the measurement, after which it began to rise steadily. After another 10 s, the fluid reached 77% of its initial viscosity, and after 30 s of the third stage of measurement, its structure was rebuilt to 81% initial viscosity. From a practical point of view, the relatively quick recovery of the STF to a near-baseline state after the sudden application of high stress is extremely important. This is because it allows us to say that protective materials based on this type of shear thickening fluid will return relatively quickly to the state at which their protective properties are the greatest, following energy dissipation, e.g., impact. At the end of the measurement—at 600 s in the third stage—the measured STF’s viscosity was 98% of the initial value.

For the second fluid, based on SF silica, a sharp drop in viscosity was recorded at 105.4 s of the measurement, with the minimal value of viscosity measured at 89 Pa·s, which was more than 4 times higher than the initial viscosity in the first stage. Subsequently, the viscosity of the system started to increase again at 164 s of the measurement (61 s after the end of the application of high stress on the sample). The viscosity of the fluid at this point was more than 17 times higher than its initial viscosity. Next, the STF’s viscosity gradually decreased, but after the next 9 min, it was still over five times higher than in the beginning. Such results may indicate that the irregular particles will probably return to their original arrangement (state) and the fluid will reach its initial viscosity. However, this process was very slow, and the required time significantly exceeded the measurement time. It should be remembered that the viscosity of suspensions containing non-spherical particles is strongly dependent on their mutual orientation, as well as on the orientation of the flow direction. Thus, the irregular silica particles are conducive to numerous, unpredictable collisions and jamming, and the course of the curve for this sample (Figure 10a) might be a confirmation of this. From a technological point of view, however, the slow reorganization of the structure and the time taken to return to the initial state after applied stress, e.g., after impact, are not advantageous, whether the viscosity is higher or lower than it is initially. For a long period of time, the material may exhibit properties other than what is required for the application for which it was designed.

**Figure 11:**
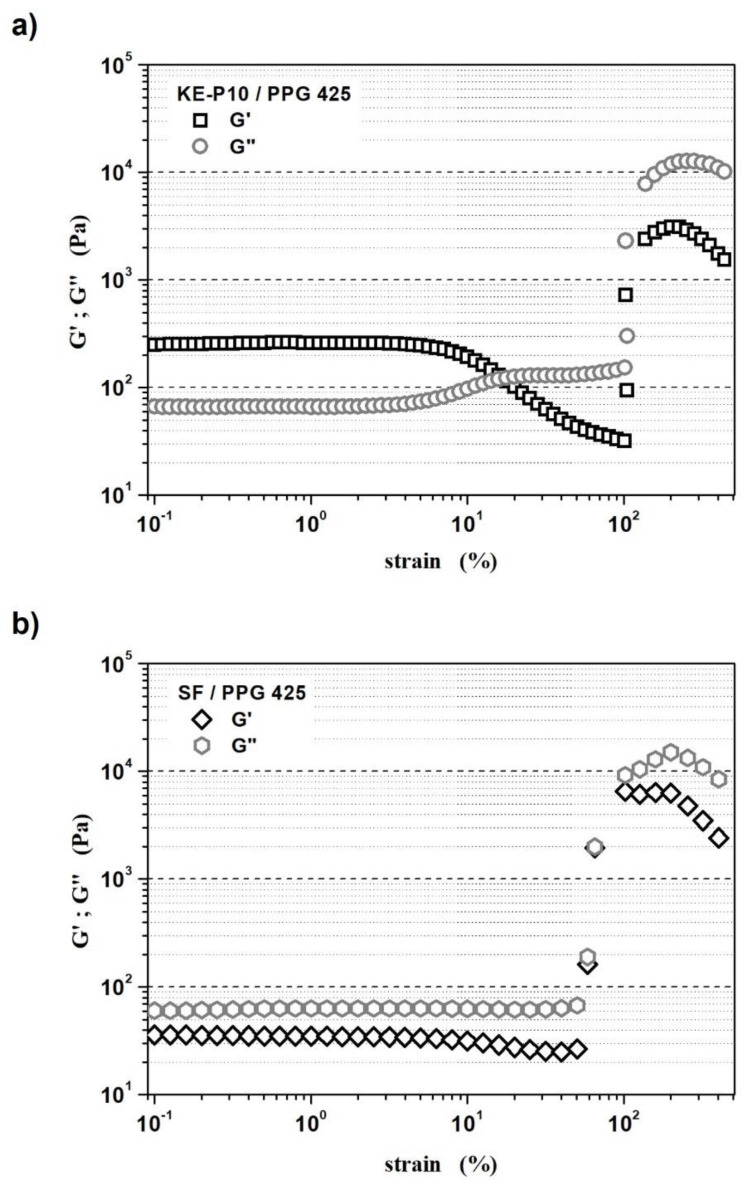
Storage modulus G′ and loss modulus G″ as a function of oscillation strain for (**a**) a shear thickening fluid based on KE-P10 silica and poly(propylene glycol) with an average molecular weight of 425, and (**b**) a shear thickening fluid based on SF silica and poly(propylene glycol) with an average molecular weight of 425. The solids contents of the fluids with KE-P10 and SF silica were 50 and 20 vol.%, respectively. The measurements were carried out at a temperature of 20 °C.

The next step toward the characterization of both fluids was to conduct oscillatory measurements. The results obtained on the basis of the performed amplitude scans (for deformation from 0.1 to 500% at a constant frequency of 1.6 Hz) (Figure 11) allowed us to determine a linear viscoelastic region (LVER):

- The LVER of the STF with KE-P10 was from 0.1 to 3.2% of oscillation strain;
- The LVER of the STF with SF was from 0.1 to 4% of oscillation strain.

In KE-P10/PPG 425 system, the storage modulus (G′) dominates over the loss modulus (G″) in the oscillation strain range of 0.1 to approx. 17%. However, above the LVER, when the oscillation strain exceeded 3.2%, the storage modulus (G′) clearly decreased, which indicates the breakdown of the material structure. For an oscillation strain greater than 100%, the G′ (and also G″) increased sharply due to the structure building up as a result of the shear thickening process.

In the SF/PPG 425 system, the loss modulus (G″) dominated over the storage modulus (G′) in the whole range of applied oscillation strain. Above the LVER, when the oscillation strain exceeded 4%, the value of G′ slightly decreased, and only between 58 and 100% were the values of G′ and G″ close to each other. For an oscillation strain above 50%, the rapid increment of the G′ and G″ moduli is observed.

**Figure 12:**
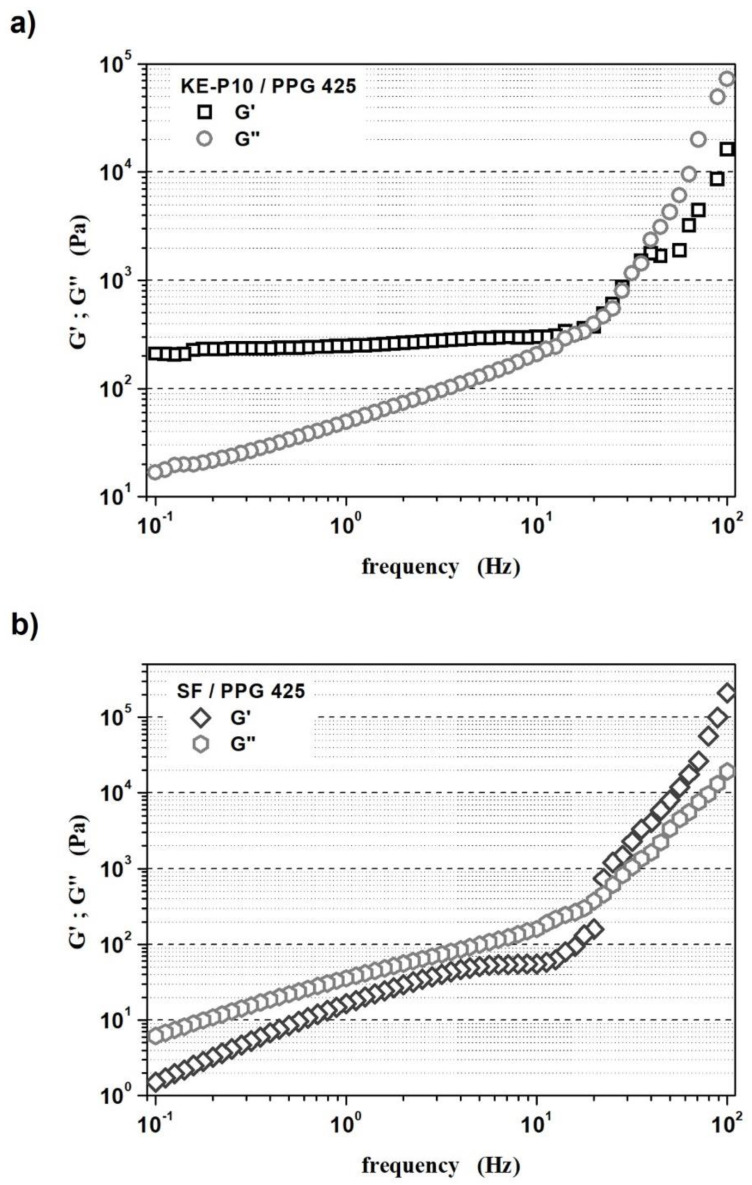
Storage modulus G′ and loss modulus G″ as a function of frequency for (**a**) a shear thickening fluid based on KE-P10 silica and poly(propylene glycol) with an average molecular weight of 425, and (**b**) a shear thickening fluid based on SF silica and poly(propylene glycol) with an average molecular weight of 425; The solids contents of the fluids with KE-P10 and SF silica was 50 and 20 vol.%, respectively. The measurements were carried out within the LVER at an oscillation strain of 1% and temperature of 20 °C.

The determination of the LVER area for both samples made it possible to perform frequency tests. These measurements were carried out at an oscillation strain of 1% for both fluids. Figure 12 shows the dependence of the G′ and G″ moduli as a function of frequency. The obtained results, i.e., the dependence of the G′ and G″ moduli as a function of frequency, are shown in Figure 12.

Figure 12a shows the domination of G′ over G″ in the frequency range from 0.1 to 15 Hz for the STF with KE-P10. This indicates an elastic response of the material to the given strain—the sample shows a gel-like structure. Moreover, the value of the storage modulus was relatively constant, while the value of the loss modulus increased over 14 times. For higher frequencies, in the range of 15–38 Hz, the values of both moduli were very similar to each other, so the viscous behavior was equal to the elastic behavior. Above 38 Hz, the viscous response of the samples was noticeably dominant. Generally, above 20 Hz the values of both moduli increase rapidly.

A different response of the fluid with SF silica to applied strain is presented in Figure 12b. In the frequency range from 0.1 to about 20 Hz, the loss modulus G″ dominated over the storage modulus G′, which shows the viscous response of the material. In the entire tested frequency range, the value of both moduli increased with higher frequency. However, above 14 Hz, the G′ increased much faster, and for frequencies above 20 Hz, the elastic properties became dominant over the viscous properties. Thus, it can be concluded that the sample containing irregular particles of SF silica as a solid phase behaves like a viscoelastic fluid.

### 3.3. Effect of Silica Morphology on the Protective Abilities of STFs

Kinetic energy dissipation tests were performed on the basis of the BS 7971-4:2002 standard. For these tests, STF–silicone composite materials were used. Despite the fact that the STF with irregular silica SF shows a 45% higher dilatant effect, and its response to applied shear stress is also greater, the average results of impact energy dissipation were slightly smaller than in the case of the fluid with KE-P10. For applied kinetic energy (5 J), the composite material with SF silica and the composite material with KE-P10 silica dissipated 59.2 ± 2.2% and 60.5 ± 3.0% of the energy of the impact, respectively. One can see that the differences in the obtained results, however, were within the limits of the standard deviation. Therefore, it cannot be stated unequivocally that any of the samples showed better or worse protection properties. It must be remembered that the composite materials were tested, thus, the obtained results are the response of composites (the combined response of the silicone and STF) and not the fluids alone.

That is why kinetic energy dissipation tests with a drop tower equipped with a rod–cup measuring system were performed. In this kind of test, only the response of fluid to the applied impact energy is analyzed, so a 10 times higher (50 J) impact energy was used. The height of the liquid column was 80 mm. On each fluid, a rod was dropped five times. Table 6 shows the results of the conducted kinetic energy dissipation tests. The registered forces for the fluids with KE-P10 and SF silica were 1.27 ± 0.02 kN and 1.37 ± 0.03 kN, respectively. As a reference fluid, water was used. In this case, the recorded force was 40.27 ± 1.16. It is worth emphasizing that the applied measurement system ensures very good repeatability of the results, which makes the obtained results very reliable. The value of the force passing through the fluid with KE-P10 silica, recorded by the sensor placed under it, was 8% lower. It shows that the protective properties of STFs containing spherical silica KE-P10 as a solid phase are slightly more favorable, despite the fact that this fluid shows a slightly worse shear thickening effect and a lower STR coefficient. Such results are quite surprising, because usually, the greater the ability of shear thickening fluids (more precisely, samples containing STFs) to dissipate kinetic energy, the better the rheological parameters [18,84]. The common opinion claims that the ability of STFs to dissipate kinetic energy increases with the enhancement of the shear thickening effect.

One of the reasons for this result may be the effect of the solid-phase concentration. It is postulated that the impact energy can be dissipated more efficiently when the maximum number of vibration transmissions between different phases is ensured. In this case, the preferred mechanism would be energy transfer and dissipation via SiO_2_–PPG–SiO_2_–PPG. In the fluid with SF silica, the volume fraction of silica is much lower than in the fluid with KE-P10, and so more energy is transmitted via PPG–PPG interactions.

However, there is no doubt that the issue of the interdependence between the composition, rheological properties, and the actual ability of the STF to dissipate kinetic energy is extremely interesting, and may bring about new and valuable knowledge. It is also not as trivial as it might have seemed before, and therefore still requires further research on a much larger group of samples.

## 4. Conclusions

Our research on the developed shear thickening fluids allows us to draw a number of conclusions:

- The rheological properties of silica–glycol systems are strongly dependent on the morphology of the particles used as the solid phase:
  - Nanosized, but highly agglomerated, silica SF with irregular shapes of particles promotes the earlier occurrence of the shear thickening (dilatant) effect, with 12.5 vol.% of silica being enough to observe the thickening effect. Furthermore, it is hardly possible to prepare an STF with more than 22 vol.% of this silica;
  - In order to create a shear thickening effect when spherical and well-dispersed KE-P10 silica is used, it is necessary to add three times more silica (40 vol.%). The maximum concentration of this silica in the fluid may be 53.75 vol%. Due to the high volume of the solid phase, the shear thickening effect is three times greater than in the case of the STF with SF silica and is equal to 29 kPa∙s;
  - The application of the irregular SF silica favors higher STR coefficients;
  - Fluids with SF silica show a different profile of viscosity curves than fluids with KE-P10;
  - The morphology of silica particles influences the reorganization of the internal structure after the shear;
  - Spherical silica facilitates reorganization and the fluid more quickly returns to its initial state;
- We propose the distinguishment of a new term—technological critical shear rate ${\dot{\gamma }}_{cr-T\;}$. From a technological point of view, ${\dot{\gamma }}_{cr-T\;}$ should be taken into account, because only after exceeding this value of shear rate does the viscosity of the system increase rapidly. When considering the practical use of shear thickening fluids, only a sudden and abrupt increase in viscosity ensures a proper response to the external stimulus applied;
- The high silica content in shear thickening fluids might enhance the formation of internal networks through hydrogen bonds that cause the domination of elastic properties over viscous properties (G′ > G″);
- The unique measurement system utilized during the kinetic energy dissipation tests allows one to measure the response of pure fluids (without their prior encapsulation or packaging), and thus obtain real and precise results;
- The results of research on the protective properties of the tested STFs show that it is not necessarily the fluids with a greater shear thickening effect as well as higher STR coefficient that can dissipate more energy and protect to a greater extent. This finding questions the legitimacy of obtaining STFs with the highest possible shear thickening effect in the context of their protective properties.

Finally, the practical aspects of the preparation of STFs must be taken into account. The preparation of a homogeneously mixed fluid is sometimes a very difficult, complicated, and long process. Working with SF silica, in particular, can be challenging, due to the very low bulk density and the dusting of this kind of SiO_2_. Moreover, the homogenization process for STF with SF silica takes 2–3 times longer than for fluids with spherical silica, which, of course, also increases energy consumption. This generates higher production costs and causes a greater environmental burden.

Taking into account the results obtained, as well as strictly technological aspects, it can be concluded that spherical silica is a more favorable raw material for shear thickening fluids. The future works of the authors will focus on the comparison of the functional properties of STFs based on spherical particles. The influence of the spherical particle size, as well as the chemical structure of the dispersing liquids on the ability to dissipate kinetic energy, will be analyzed.

## Figures and Tables

**Table 1:** Influence of selected factors on the rheological properties of shear thickening fluids.

Factor	Influence on the Shear Thickening Phenomenon
Solid-phase content	The increase in the volume fraction of the solid phase reduces the critical shear rate and enhances the shear thickening effect. The reason for this is the reduction in the distance between solid-phase particles, and hence an increase in hydrodynamic forces [4,10,18,36,46,62,63].
Particle shape of solid phase	The increase in the shape factor enhances the shear thickening effect and reduces the critical shear rate. When using particles of irregular shape, the phenomenon of shear thickening occurs at a lower volume fraction in the system [4,36].
Particle size of solid phase	The decrease in the particle size of the solid phase usually increases the critical shear rate. The reason for this is the domination of Brownian forces for nanometric particle suspensions [4,64,65]. The application of smaller particles often results in a higher viscosity of the STF and a greater shear thickening effect [45,66].
Particle hardness of solid phase	The increase in hardness of the solid phase particles is advantageous in achieving an enhanced shear thickening effect at high stress. Particles with lower hardness (e.g., PMMA) can deform under such conditions, leading to a reduction in the shear thickening phenomenon [67].
Molar mass of carrier fluid	The increase in the molar mass of the carrier fluid increases the viscosity of the STF, enhances the shear thickening effect, and reduces the critical shear rate [32,36,41,44,68,69].

**Table 2:** Physicochemical characterization of the silica powders.

Symbol	Producer	Based on the Specification Provided by the Producer				Based on Own Research				
		Specific Density [g/cm^3^]	Bulk Density [g/cm^3^]	Particle Size [nm]	Specific Surface Area [m^2^/g]	Specific Density [g/cm^3^]	Bulk Density [g/cm^3^]	Average Particle Size (DLS Method) [nm]	Average Particle Size (SEM Method) [nm]	Specific Surface Area [m^2^/g]
**KE–P10**	Nippon Shokubai, Japan	2.0	0.2	100–200	no data	1.96	0.33	127	126	114
**SF**	Sigma-Aldrich, USA	no data	0.04	200–300 agglomerates	175–225	1.53	0.04	218	-	179

**Table 3:** Physicochemical characterization of the carrier fluid.

Carrier Fluid	Symbol	Producer	Based on the Specification Provided by the Producer				Based on Own Research			
			Number Average Molecular Weight M_n_	Specific Density [g/cm^3^]	Viscosity [Pa∙s]	Hydroxyl Value [mg KOH/g]	Number Average Molecular Weight of the Major Series M_n_	Weight Average Molecular Weight of the Major Series M_w_	Dispersity	Viscosity [Pa∙s]
**poly(propylene glycol)**	PPG 425	Sigma-Aldrich, USA	425	1.004	0.08	263	498	517	1.04	0.08

**Table 4:** Rheological behavior of fluids made from SF or KE-P10 silica and poly(propylene glycol) with an average molecular weight of 425. Rheological measurements were carried out at 20 °C.

	Silica	KE-P10	SF
Solid Loading (vol.%)			
10.0		X	Newtonian-like behavior
12.5		X	slight shear thickening
15.0		X	slight shear thickening
17.5		X	significant shear thickening
20.0		shear thinning	significant shear thickening
22.0		shear thinning	significant shear thickening
23.0		shear thinning	not possible to obtain
25.0		shear thinning	not possible to obtain
30.0		shear thinning	X
35.0		shear thinning	X
40.0		slight shear thickening	X
45.0		shear thickening	X
47.5		shear thickening	X
50.0		significant shear thickening	X
52.5		significant shear thickening	X
53.75		significant shear thickening	X
55.0		homogeneus fluid	X
56.25		not possible to obtain	X

**Table 5:** Results of the rheological measurements of shear thickening fluids made of KE-P10 spherical silica or SF fumed silica and poly(propylene glycol) with an average molecular weight of 425.

Composition	Solid Loading (vol.%)	Initial Viscosity (Pa∙s)	Beginning of Shear Thickening		Maximum Viscosity		Shear Thickening Effect (Pa∙s)	Shear Thickening Ratio
			Shear Rate (s^−1^)	Viscosity (Pa∙s)	Shear Rate (s^−1^)	Viscosity (Pa∙s)		
**KE-P10/PPG 425**	50.0	119.5	5.8	22.1	14.7	1590	1568	71.9
**SF/** **PPG 425**	20.0	20.4	0.4/1.3 *	21.5	5.9	2296	2275	106.8

* technological critical shear rate.

**Table 6:** The results of impact energy dissipation tests using a rod–cup system for shear thickening fluids made of KE-P10 spherical silica or SF silica fumed and poly(propylene glycol) with an average molecular weight of 425.

Composition	Solid Loading (%)	Impact Energy (J)	STF Temperature (°C)	Force (kN)
Water (reference)	-	50	20.0	40.27 (±1.16)
KE-P10/PPG 425	50.0	50	20.1	1.27 (±0.02)
SF/PPG 425	20.0	50	20.0	1.37 (±0.03)

## Data Availability

Data sharing not applicable.
